# Meal Timing, Meal Frequency and Metabolic Syndrome

**DOI:** 10.3390/nu14091719

**Published:** 2022-04-21

**Authors:** Fatema Alkhulaifi, Charles Darkoh

**Affiliations:** 1 Department of Epidemiology, Human Genetics & Environmental Sciences, School of Public Health, University of Texas Health Science Center at Houston, Houston, TX 77030, USA; fatema.alkhulaifi@uth.tmc.edu; 2 Microbiology and Infectious Diseases Program, Graduate School of Biomedical Sciences, University of Texas MD Anderson Cancer Center UTHealth, Houston, TX 77030, USA

**Keywords:** meal timing, meal frequency, skipping meals, fasting, obesity, metabolic syndrome, diabetes

## Abstract

Individuals with metabolic syndrome have increased risk for developing health conditions, including cardiovascular diseases and stroke. Modifiable risk factors, such as exercise and diet, are key components in the prevention and control of metabolic syndrome. Specifically, dietary patterns and habits are extremely successful in controlling more than one of the metabolic syndrome risk factors. Meal timing and frequency have been associated with type 2 diabetes, cardiovascular diseases, and other chronic conditions. However, there is limited evidence linking metabolic syndrome to meal timing and meal frequency. This review summarizes and discusses how meal timing and frequency impact metabolic outcomes in adults.

## 1. Introduction

An estimated 20–25% of adults worldwide have metabolic syndrome [1]. The most rapid increase in metabolic syndrome is seen in the urban population of developing countries. Nonetheless, metabolic syndrome is increasing among United States (US) adults with the rates rising from 32.5% in 2011 to 36.9% in 2016 [2]. Moreover, 12–26% of adults in European countries are diagnosed with metabolic syndrome [3]. Individuals with metabolic syndrome have a two-fold greater risk of dying and a three-fold greater risk of having a heart attack or stroke compared to individuals without metabolic syndrome [1]. Furthermore, individuals with metabolic syndrome are five times as likely to develop type 2 diabetes [4]. As the burden of metabolic syndrome increases, it is important to understand which lifestyle factors, such as diet and physical activity, are central to developing metabolic syndrome.

Diet plays an important role in the prevention and management of obesity, diabetes, and cardiovascular diseases. As research in recent decades has focused more on dietary patterns, control of metabolic syndrome has been correlated with them. Healthier dietary patterns have been associated with a lower risk of metabolic syndrome, while unhealthy patterns are associated with a higher prevalence of metabolic syndrome [5]. Modifying the diet to control metabolic syndrome is a desirable approach, as the collection of risk factors that contribute to metabolic syndrome are all affected by food. Furthermore, encouraging individuals to follow a food-based approach to control metabolic syndrome can lead to improving the home eating environment [6].

As scientific evidence is leaning towards eating behaviors in addition to healthy consumption of specific nutrients, different elements of the diet are explored. In the past five years, research demonstrated that meal timing and meal frequency are associated with multiple chronic diseases. Skipping breakfast, lunch, or dinner has become prominent today as more people eat outside their homes [7,8]. In addition, epidemiological studies have shown that eating late at night is associated with an increased risk for obesity [9] and cardiovascular disease [10,11]. Moreover, newfound evidence suggests that meal timing and meal frequency are associated with metabolic syndrome in numerous ways.

It is challenging to specify which one element of the diet is responsible for metabolic syndrome risk factors. Nonetheless, understanding the variety in the human diet will help understand how to prevent and control chronic conditions such as metabolic syndrome. This review aims to evaluate the evidence on the association between meal timing and meal frequency, and metabolic syndrome in adults. Four main dietary topics are reviewed for their association with metabolic syndrome: meal time, meal frequency, skipping meals, and fasting.

## 2. Risk Associated with Metabolic Syndrome

The presence of metabolic syndrome, by the occurrence of multiple risk factors in an individual, has been associated with many noncommunicable chronic diseases [12]. Although there are many definitions of metabolic syndrome, and they differ slightly, there is consensus that a person with metabolic syndrome presents with three or more risk factors. The risk factors include elevated blood pressure, high fasting blood glucose, large waist circumference, increased triglycerides (TG) in the blood, and reduced levels of high-density lipoprotein (HDL) in the blood. All metabolic syndrome risk factors have been linked with heart disease, diabetes, stroke, and other health problems [13]; nonetheless, the risk of metabolic syndrome is reduced significantly by losing weight, increasing physical activity, and consuming a healthy diet.

Wannamethee [14] evaluated the British Regional Heart Study for risk associated with metabolic syndrome and demonstrated that metabolic syndrome significantly increases the risk of coronary heart disease (relative risk [RR] = 1.57, 95% Confidence Interval [CI] = 1.39–1.97), stroke (RR = 1.61, 95% CI = 1.26–2.06), and type 2 diabetes mellitus (RR = 3.57, 95% CI = 2.38–4.50) [14]. Furthermore, in the Atherosclerosis Risk in Communities Study, McNeill et al. [15] revealed that metabolic syndrome is associated with cardiovascular disease (CVD) morbidity over a mean study period of 11 years. The authors of the study concluded that men and women with metabolic syndrome were 1.46 (95% CI: 1.23–1.74) and 2.05 (95% CI: 1.59–2.64) times more likely to develop coronary heart disease than those without metabolic syndrome, after controlling for age, smoking, low-density lipoprotein (LDL) cholesterol, and race [15]. A study with a similar follow-up time showed that men with metabolic syndrome had a significantly increased risk of death due to coronary heart disease (RR = 2.9 to 4.2) after adjusting for the usual cardiovascular risk factors.

Metabolic syndrome is associated with other chronic conditions such as spinal osteoarthritis. To evaluate the increased risk of spinal osteoarthritis, Gandhi et al. [16] assessed the prevalence of metabolic syndrome among patients with severe spinal osteoarthritis. The risk factors of metabolic syndrome were more predominant in patients diagnosed with severe spinal osteoarthritis compared to those diagnosed with early osteoarthritis. The metabolic syndrome risk factors (waist circumference, cholesterol, fasting glucose, and blood pressure) were associated with nearly quadruple the odds of having severe spinal osteoarthritis compared with the absence of the risk factors [OR = 3.9 (1.4–11.6, *p* < 0.01] [16].

Since metabolic syndrome increases the risk of multiple chronic diseases, it is essential to understand the association between metabolic syndrome and lifestyle factors. At first, findings from the literature have demonstrated that the development of metabolic syndrome is the aftermath of people’s calorie consumption, when it is disproportionately high compared to their metabolic requirement. However, it has become evident during the past 10 years that numerous factors contribute to the development of metabolic syndrome.

## 3. Lifestyle Factors Affecting Metabolic Syndrome

**Figure 1:**
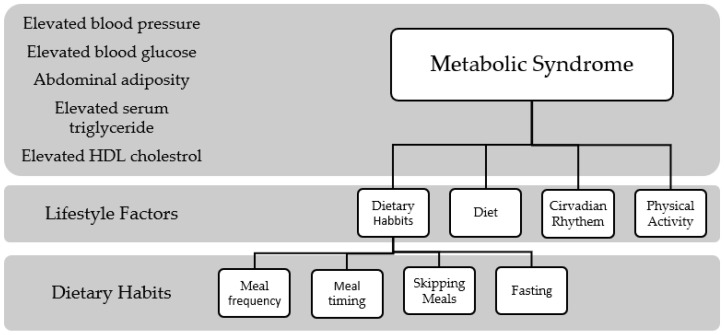
The relationship between Metabolic Syndrome and Lifestyle Factors.

To understand the association between diet and metabolic syndrome, it is essential to shed light on the role of lifestyle factors in metabolic syndrome. Abdominal obesity, the risk factor present in every definition of metabolic syndrome, has been linked to multiple lifestyle choices such as lack of physical activity and a Western diet. Moreover, sleep or lack thereof has been linked with metabolic syndrome risk factors such as increased blood pressure [17] and insulin resistance [18]. As illustrated in Figure 1, many lifestyle factors contribute to the development of metabolic syndrome. Understanding the mechanism of how these risk factors are associated with metabolic syndrome is important to be able to control the syndrome worldwide.

### 3.1. Role of Physical Activity on Metabolic Syndrome

The benefits of physical activity include sustaining a healthy weight, improving mental health, quality of life, and well-being. Physical activity, defined as all body movements, has been proven to prevent and help manage non-communicable diseases, including heart disease, stroke, diabetes, and some cancers [19]. More importantly, high levels of physical inactivity or sedentary behavior have been associated with adverse outcomes on health. For adults, recommendations include 150–300 min of moderate-intensity aerobic physical activity per week or 75–150 min of vigorous-intensity aerobic physical exercise per week [19]. Around the world, about 25% of adults do not meet the recommended guidelines for physical activity [19].

Physical activity is associated with a decreased risk of metabolic syndrome [20,21]. As the prevalence of metabolic syndrome has increased, total physical activity expenditure has decreased during the same period [22]. Rennie et al. [20] conducted a study of 5153 European adults that assessed the association between physical activity and the prevalence of metabolic syndrome. The results showed that an increase in activity of moderate (OR = 0.78, 95% CI = 0.63–0.96) and vigorous physical exercise (OR = 0.52, 95% CI = 0.40–0.67) is associated with lower odds of metabolic syndrome after controlling for age, sex, smoking, alcohol intake, socioeconomic status, and other activity [20]. Other intervention studies have shown that physical activity is effective in decreasing body weight and visceral fat accumulation [23,24], controlling blood pressure [25], improving HDL cholesterol and triglycerides [25,26], and improving insulin sensitivity [23].

On the contrary, physical inactivity is associated with an increased risk for metabolic syndrome. A study by Ford et al. [21] demonstrated that metabolic syndrome is twice as likely to be present in those who do not engage in any moderate or vigorous leisure-time physical activity (OR = 1.9, 95% CI = 1.22–2.97) compared to those who engage in ≥150 min per week of moderate or vigorous leisure-time physical activity [21]. Furthermore, it was found that those who have an increased sedentary behavior ≥4 h per day have an increased risk for getting metabolic syndrome after controlling for age, sex, race or ethnicity, educational status, smoking status, and alcohol use (OR = 2.10, 95% CI = 1.27–3.47) [21]. Hence, physical activity can play an important role in decreasing the prevalence of metabolic syndrome and, ultimately, chronic conditions.

### 3.2. Sleep, Circadian Rhythm, and Metabolic Syndrome

Within the last 10 years, interest in circadian rhythms has grown tremendously. Circadian rhythms are defined as physical, mental, and behavioral changes that fall within a 24-h cycle [27]. Evidence shows that circadian rhythms affect the human body in many ways, including hormone release, eating habits and digestion, temperature, and sleep pattern. Disorders of circadian rhythms include health issues that appear when the sleep-wake cycle is not aligned correctly with the environment and hinders usual activities. Circadian disorders are the results of environmental components that affect circadian rhythms. Some of the major features that affect circadian rhythms are light, nutritional intake, and weather [28].

A link has been established between circadian rhythms and components of metabolic syndrome, such as glucose metabolism [29] and obesity [30]. Furthermore, circadian rhythm is associated as a contributor to major chronic diseases such as type 2 diabetes [29] and CVD [31]. Since metabolic syndrome has a substantial socio-economic impact on most developing countries, examining a possible link to circadian rhythms is essential to understand the association further. It is possible that in communities that experience more night eating and less sleep at night, the circadian rhythm is more prominent. Night light, nutritional intake, and weather are vital contributors to circadian rhythm [2,28]. It is essential to investigate how night eating, mealtime, and frequency affect metabolic syndrome in a place that experiences a more lively, light-filled nightlife.

### 3.3. Diet and Metabolic Syndrome

Diet quality and dietary patterns have been associated with various chronic conditions, including heart disease, cancer, and diabetes [5,6]. A study in China by He et al. conducted on 35,146 participants from China National Nutrition and Health Survey revealed that high diet quality is associated with a lower risk [OR = 0.79 (95% CI: 0.69–0.91)] of metabolic syndrome [32]. The study utilized the Global Diet Quality Score to measure diet quality by analyzing how much of the diet belongs to healthy and unhealthy food groups. Dietary patterns, such as the Mediterranean diet, have been associated with metabolic syndrome. A meta-analysis by Kastorini et al. [33] revealed that the Mediterranean diet was associated with a lower risk of metabolic syndrome (log Hazard Ratio (HR) = −0.69, 95% CI −1.24 to −1.16) [33]. Since diet quality or dietary patterns do not fully explain the association between diet and metabolic syndrome and its risk factors, interest has grown in recent years about the effect of dietary habits on metabolic syndrome prevalence.

## 4. Dietary Habits and Metabolic Syndrome

Epidemiological studies have shown that dietary habits are as important as nutrients in determining health outcomes [5,34]. There are a variety of dietary habits that are associated with the prevalence of metabolic syndrome and related conditions. Whether the practice increases the odds or decreases the odds of metabolic syndrome, it is essential to understand how our dietary habits play a role in developing different diseases and conditions (Table 1).

### 4.1. Meal Timing

While there is consensus across cultures on what a meal is, i.e., a certain amount of food eaten at a specific time, different cultures across the world follow different meal schedules. In some cultures, snacking at night is more predominant, while others snack more during the day. Moreover, weather can be one of the determinants of a person’s meal schedule. Individuals living in hot, humid weather climates may experience more nighttime eating because they tend to leave their house at night to visit friends and family. Thus, meal timing is an important determinant of their health. For instance, the tendency to eat meals at certain times of the day is associated with metabolic syndrome in different ways, depending on the time of the meal [9,37].

Ha et al. [37] reported in a cross-sectional study of meal timing and frequency in Korean adults that eating in the morning was associated with a reduced prevalence of metabolic syndrome in men (OR = 0.73, 95% CI: 0.57–0.93) and women (OR = 0.69, 95% CI: 0.54–0.89) compared to not eating in the morning [37]. Moreover, the study showed that only eating at night was significantly associated with a higher prevalence of metabolic syndrome in men (OR = 1.48, 95% CI: 1.15–1.90) than not eating at night. On the other hand, a longitudinal study by Yoshida et al. [9] showed that women with night eating habits had higher odds of developing metabolic syndrome at follow-up than those without night eating habits (OR: 1.68; 95%CI = 1.00–2.84), after adjusting for age, smoking, alcohol, physical activity, and breakfast intake [9]. The study assessed night eating in 8153 adults aged 40–54 years over approximately 4 years. Women with night-eating habits have twice the odds of obesity compared with those without these habits [(OR: 2.11; 95% CI = 1.42–3.15) for men and (OR: 3.02; 95% CI = 1.72–5.29)] [9].

Overall, there seems to be a trend that night eating is associated with an increased prevalence of metabolic syndrome while eating in the morning has a protective effect on metabolic syndrome. Nonetheless, there is a limited number of studies on night eating and metabolic syndrome, and more research is needed to fully understand the association.

### 4.2. Meal Frequency

Meal frequency is another habit that has gained attention in recent years. There seems to be an association between the number of meals consumed every day and chronic diseases. Holmbäck et al. [34] demonstrated that eating more frequently, six or more meals per day, is associated with a reduced rate of obesity in adults compared with eating less frequently, less than three meals per day [34]. The researchers utilized a subsample of the Malmo Diet and Cancer Study by including middle-aged adults between the ages of 47 to 68 years and calculating meal frequency based on self-reported eating episodes. Also, a study by Titan et al. [35] assessed the association between meal frequency and metabolic syndrome risk factors. The population-based cross-sectional study used data from the European Prospective Investigation into Cancer (EPIC) project. The findings indicated a lower concentration of total and LDL cholesterol, a difference of 0.25 mmol/L, in individuals who consumed more than six meals per day compared with individuals who consumed one or two meals per day [35]. Even after controlling for age, body mass index (BMI), physical activity, smoking, total energy intake, and macronutrient distribution, the association was strong.

The frequency of meals seems to affect blood glucose levels, as higher fasting glucose levels are one of the risk factors of metabolic syndrome. Carlson et al. [39] conducted a randomized cross-over trial over eight weeks of in-treatment diet and eleven weeks off-treatment. Fifteen subjects were randomized into either one meal or three meals per day. Individuals with only one meal had higher fasting plasma glucose levels and impaired morning glucose tolerance compared to those eating three meals per day [39]. Furthermore, a study by Stote et al. [38] that restricted meals to one meal per day for eight weeks in healthy adults resulted in a reduction in their weight, but no apparent difference in the levels of serum lipids, glucose or insulin between them and those who consumed three meals per day [38]. Additional investigation into the association will be necessary to further understand the effects of meal timing and frequency on diabetes, obesity, and metabolic syndrome.

### 4.3. Skipping Meals

Eating meals irregularly is a common practice given the fast pace of society and irregular work schedules. A recent survey of Americans in the workplace reported that approximately 60% of millennials, born between 1980 and 1995, skip lunch to get ahead of work [7]. Another survey conducted in 2011 indicated that one out of ten Americans skips breakfast [8]. Skipping breakfast was seen commonly in males and those between the ages 18–34 years [8]. A systematic review of 35 articles revealed that breakfast is the meal most frequently skipped, followed by lunch and then dinner [47]. With skipping meals being common, it is important to investigate whether it is associated with health risks, specifically common risk factors such as those related to metabolic syndrome.

Eating irregularly and frequently skipping meals is less favorable for attaining a healthy cardiometabolic profile [48]. In general, skipping breakfast or lunch has a more significant impact on the diet than skipping dinner [49]. Furthermore, skipping breakfast has been linked with excess body weight and insulin resistance [50]. Skipping breakfast has also been associated with obesity and mental health problems in children and adolescents [51,52]. A cohort study by Laguzzi et al. [40] in Sweden that followed 60-year old men and women for up to 20 years revealed that eating irregular meals was associated with an increase in the risk of CVD in men (Hazard Ratio (HR) = 1.70, 95% CI: 1.19–2.43) after controlling for civil status [40].

Skipping meals is also associated with metabolic syndrome. As shown by Sierra-Johnson et al. [41] in a cross-sectional study, those who did not eat regularly were significantly more likely to have metabolic syndrome compared with those who ate regularly [41]. The authors of the study found that those who have the greatest number of metabolic syndrome components have a lower incidence of eating regular meals (OR = 0.27, 95% CI: 0.13–0.54) compared with those who do not have any of the components of metabolic syndrome. Additionally, eating regular meals was significantly inversely associated with insulin resistance (OR = 0.68, 95% CI 0.48–0.97) [41]. Chung et al. [53] illuminated in a cross-sectional study of Korean adults that individuals who skipped breakfast were more likely to have the lowest intake of Korean Recommended Nutrient Intake levels and increased odds for metabolic syndrome (OR = 1.2, 95% CI 1.04–1.38) [53]. A prospective cohort study by Wennberg et al. [42] found that adolescents who ate irregular meals are associated with metabolic syndrome in their adult life. The Swedish study followed individuals for 27-years and showed that eating meals irregularly at 16 years was associated with metabolic syndrome at 43 years (OR = 1.74, 95% CI: 1.12–2.71) [42]. Eating irregular meals seems to be associated with some adverse effects and conditions; however, there is still not enough evidence to fully understand the association. Further research is needed to determine the extent to which irregular eating habits adversely affect health.

### 4.4. Fasting

Dawn-to-sunset fasting is one of the most commonly practiced types of fasting across the world. Even though this type of fasting is mostly practiced for religious reasons, there seems to be health benefits associated with it. Dawn-to-sunset fasting affects different metabolic syndrome risk factors. Al-Shafei [44] revealed in a prospective study of 80 adults that dawn-to-sunset fasting during the month of Ramadan led to a significantly lower serum triglyceride (22.1%), lower malondialdehyde (46.6%), and an increase in HDL (6.7%) [44]. Another study of 81 healthy adults by Ziaee et al. [36] showed a significant decrease in BMI during fasting, but no benefit was seen in their lipid profile [36]. Another prospective study that measured anthropometric, metabolic, and endocrine parameters in 27 healthy adults three days before Ramadan and three days before the end of Ramadan showed a significant (<0.01) decrease in body weight, BMI, waist circumference, and body fat [43].

Many are choosing to fast at different times of the day since dietary fasting is not limited to dawn to sunset. Intermittent fasting is gaining popularity as a healthy method of losing fat and maintaining a desirable weight. In a clinical trial, Anton et al. [46] used time-restricted feeding for four-weeks on older adults (>65 years) with metabolic syndrome and impaired mobility. There was no significant change in fasting blood glucose and blood pressure, but a decline in body weight was notable in the participants [46]. Another clinical trial by Guo et al. [45] recruited 39 patients with metabolic syndrome in a community center and put them on an intermittent fasting diet for a period of eight weeks where they fasted for two days a week. After the eight weeks, there was a significant decrease in weight (−3.5 kg, <0.001) in those who fasted compared to those who did not [45]. On the other hand, another report which examined different types of intermittent fasting regimens (alternate-day-fasting and time-restricted feeding) concluded that most studies on this topic were substandard and utilized a small number of participants. Thus, there is a need for further investigation on the effect of fasting on metabolic syndrome [54].

## 5. Conclusions

There are a lack of studies that explore the association between various dietary habits and metabolic syndrome. Nonetheless, current evidence has indicated that meal timing and frequency, skipping meals, and fasting are all associated with metabolic syndrome. Eating frequent meals and eating in the morning may have a protective effect on metabolic syndrome. However, eating at night, skipping breakfast, eating one meal per day, and eating irregularly may facilitate the development of metabolic syndrome risks in adults. The effects of fasting on metabolic syndrome prevalence are unclear. Understanding the effect of eating habits is as important as understanding the effect of nutrients on health. Further research is needed to understand the association between dietary habits and the development of metabolic syndrome.

## Figures and Tables

**Table 1:** Dietary habits and development of metabolic syndrome and other related conditions.

Dietary Habits	Implications in Metabolic Syndrome Development and Other Related Conditions
Eating more frequent meals	Increased rate of obesity in adults [35] Lower LDL in adults consuming >six meals [36] Inversely associated with poor glycemic control and high cholesterol
Eating in the morning	Reduced prevalence of metabolic syndrome in Korean adults [37]
Eating at night	Higher prevalence of metabolic syndrome in men [38] Increased rate of obesity [9] Increased risk of metabolic syndrome in women [9]
One meal per day	Weight loss but no benefit to lipid profile [39] Increase in fasting serum blood glucose [40]
Skipping breakfast	Excess body weight and insulin resistance [41] Correlated with obesity in adults and children [42] Correlated with mental health problems in adolescents in Korea [43] Increased odds of metabolic syndrome [43] Low intake of recommended nutrient intake [44]
Eating irregular meals	Increase in risk of CVD in men [45] Increased prevalence of metabolic syndrome [41] Increased odds of metabolic syndrome in adulthood [42]
Dawn-to-Sunset Fasting (Ramadan)	Decrease in waist circumference [43] Experience weight loss while caloric intake is the same as a non-fasting day [36] Improved lipid profile in healthy adults [44]
Intermittent fasting	Decline in weight [45,46]

## Data Availability

Not applicable.
